# Analysing the Progression Rates of Macular Lesions with Autofluorescence Imaging Modes in Dry Age-Related Macular Degeneration

**DOI:** 10.4274/tjo.93276

**Published:** 2015-12-05

**Authors:** Kenan Olcay, Akın Çakır, Murat Sönmez, Eyüp Düzgün, Yıldıray Yıldırım

**Affiliations:** 1 Gümüşsuyu Military Hospital, Clinic of Ophthalmology, İstanbul, Turkey; 2 Gölcük Military Hospital, Clinic of Ophthalmology, Kocaeli, Turkey; 3 Gülhane Military Medical Academy, Haydarpaşa Training and Research Hospital, Clinic of Ophthalmology, İstanbul, Turkey

**Keywords:** age-related macular degeneration, blue-light fundus autofluorescence, near-infrared autofluorescence

## Abstract

**Objectives:**

In this study we aimed to compare the sensitivity of blue-light fundus autofluorescence (FAF) and near-infrared autofluorescence (NI-AF) imaging for determining the progression rates of macular lesions in dry age-related macular degeneration (AMD).

**Materials and Methods:**

The study was designed retrospectively and included patients diagnosed with intermediate and advanced stage dry AMD. Best corrected visual acuities and FAF and NI-AF images were recorded in 46 eyes of 33 patients. Lesion borders were drawn manually on the images using Heidelberg Eye Explorer software and lesion areas were calculated using Microsoft Excel software. BCVA and lesion areas were compared with each other.

**Results:**

Patients’ mean follow-up time was 30.98±13.30 months. The lesion area progression rates were 0.85±0.93 mm2/y in FAF and 0.93±1.01 mm2/y in NI-AF, showing statistically significant correlation with each other (r=0.883; p<0.01). Both imaging methods are moderately correlated with visual acuity impairment (r=0.362; p<0.05 and r=0.311; p<0.05, respectively). In addition, larger lesions showed higher progression rates than smaller ones in both imaging methods.

**Conclusion:**

NI-AF imaging is as important and effective as FAF imaging for follow-up of dry AMD patients.

## INTRODUCTION

Age-related macular degeneration (AMD) is a complex disease with many genetic and environmental factors affecting its etiology, and AMD has become the most common cause of legal blindness in developed countries.^1,2,3^ AMD occurs in two forms: neovascular (wet, exudative) and non-neovascular (dry, atrophic). Wet AMD is the less common form, but causes more vision loss than dry AMD.^4^ Fundus photography, fluorescein angiography, indocyanine green angiography (ICGA) and optical coherence tomography (OCT) are among the many methods that have been introduced for the diagnosis and follow-up of AMD. However, fundus autofluorescence (FAF) imaging has also become prominent in AMD diagnosis and follow-up, and some recent studies have demonstrated its importance in helping determine course of treatment.^5,6,7,8^ In this study, we aimed to use blue-light FAF and near-infrared autofluorescence (NI-AF) images obtained from AMD patients during follow-up to monitor the progression and determine the growth rates of macular lesions, to evaluate the association between lesion progression rate and visual acuity loss, and to thereby evaluate the value of NI-AF imaging in AMD.

## MATERIALS AND METHODS

The study included 46 eyes of 33 patients who were diagnosed with moderate to severe dry AMD in our outpatient clinic between 2008 and 2013, were followed for at least 6 months, and from whom autofluorescence images were obtained during examinations within specific periods.

The records of patients with a history of any of the following were excluded from the study: pars plana vitrectomy, detachment surgery, cryopexy, argon laser photocoagulation, penetrating or blunt ocular trauma, inflammatory ocular pathologies, glaucoma, glaucoma surgery, retinal vascular pathologies (vein occlusion, diabetic/hypertensive retinopathy, etc.), complicated cataract surgery, choroidal neovascular membrane development for reasons other than AMD (high myopia, trauma, angioid streaks, etc.) and media opacity. Patients were also excluded if clear autofluorescence images could not be aquired for reasons such as non-compliance.

Patients’ best corrected visual acuity (BCVA) was calculated as logMAR (logarithm of the minimum angle of resolution).

All patients received micronutrition supplementation therapy due to their moderate to severe dry AMD.

An HRA2 angiography device (Heidelberg Retinal Angiography, Dossenheim, Germany) was used to obtain FAF images. After instilling 1% tropicamide and achieving sufficient midriyasis, FAF images were obtained using the device’s FA mode (excitation wavelength=488 nm; emission wavelength=500 nm) and NI-AF images were obtained using the device’s ICGA mode (excitation wavelength=787 nm; emission wavelength=800 nm).

**Figure 1:**
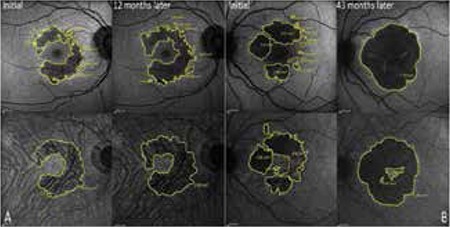
Lesions marked on fundus autofluorescence (upper row) and near-infrared autofluorescence (lower row) images acquired during follow-up. A: Fundus autofluorescence and near-infrared autofluorescence images and lesion area measurements of a patient followed for 12 months. B: Similar lesion visualization and area measurement of a patient followed for 43 months

All outlining was done by the same observer. Areas were measured in millimeters squared. These measurements were transferred to Microsoft Excel; initial and final lesion areas were calculated and recorded together with visual acuity (Figure 1).

The study was approved by the GATA Haydarpaşa Training Hospital Non-Invasive Clinical Trials Ethics Committee (02 May 2013, ethics committee number 1491-43-13). In addition, it was ensured that the patients’ records contained informed consent forms specifying that the patients had received detailed information explaining all tests prior to examination.

Lesion areas at the beginning and end of the follow-up period measured from NI-AF and FAF images were compared within and between the two imaging methods, and the correlation between these values and visual acuity changes was statistically analyzed. 

Statistical analyses were done using NCSS (Number Cruncher Statistical System) 2007 and PASS (Power Analysis and Sample Size) 2008 statistical software (Utah, USA). Spearman’s correlation analysis was used to evaluate relationships between parameters. The results were evaluated with 95% confidence interval. P values less than 0.05 were considered significant.

## RESULTS

The ages of the study participants ranged from 59 to 91 years with a mean of 79.45±7.22 years. Mean follow-up time was 30.98±13.30 months (range, 6-54 months) with a median of 27 months.

**Figure 2:**
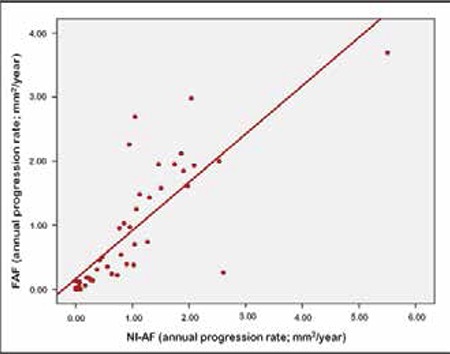
The correlation between annual progression rate on fundus autofluorescence and near-infrared autofluorescence

Median FAF lesion area growth rate was 0.42 (0.85±0.93) mm2/year, while median NI-AF lesion area growth rate was 0.78 (0.93±1.01) mm2/year. Annual growth rates from the two imaging methods showed a highly significant positive association (r=0.883; p<0.01) (Figure 2).

The median annual change in LogMAR visual acuity was 0.07 (0.086±0.016); this change was highly statistically significant (p<0.01). The same calculation based on the Snellen chart resulted in a median annual decrease of -0.06 (-0.08±0.07) in BCVA.

**Figure 3:**
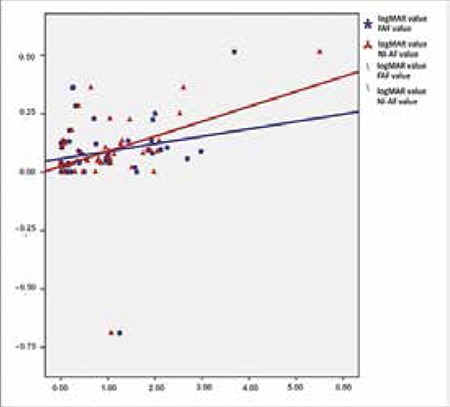
The correlation between annual best corrected visual acuity changes (LogMAR) and lesion growth rates on fundus autofluorescence and near-infrared autofluorescence

There were statistically significant positive associations between mean annual change in LogMAR BCVA values and both FAF lesion area growth rates (r=0.311; p<0.05) and NI-AF lesion area growth rates (r=0.361; p<0.05). While lesion growth was proportional to visual acuity loss, NI-AF imaging seems to be slightly more significantly associated with loss of visual acuity (Figure 3).

Another finding of our study is the effect of initial lesion size on prognosis. With both imaging methods, a statistically significant relationship emerged between initial lesion size and annual decline in BCVA (p<0.01). Patients with an initial lesion size of 2.6 mm2 (approximately 1 disc area) or larger had significantly greater changes in LogMAR BCVA values (Table 1).

Initial lesion area appears to be associated not only with visual acuity, but also with annual lesion growth rate. Patients with an initial lesion size of 2.6 mm2 or larger had significantly faster annual growth according to both FAF and NI-AF measurements (Table 1).

## DISCUSSION

FAF is an imaging technique that allows lipofuscin-based visualization of the retinal pigment epithelium (RPE), and is an effective imaging method currently in use for determining patients who are at risk based on lesion appearance, size and extent. Among measurements taken from FAF images, high-risk factors with an impact on disease progression include rapid atrophy enlargement in patients with geographic atrophy; rapid atrophy enlargement in the fellow eye; large initial atrophy size; an area of increased fluorescence surrounding the geographic atrophy; and fluorescence pattern surrounding the area of atrophy.^9,10^

With the recent discovery of the fluorescent properties of melanin, which is another fluorofor in the RPE and is as important as lipofuscin in the pathophysiology of AMD, NI-AF has opened new doors in the assessment of the RPE.^5,11^ It has also been reported that NI-AF images can reveal different lesion characteristics than conventional FAF images.^5^

Macular lesions in AMD can present as dots or lines with increased or decreased autofluorescence in FAF or NI-AF, and can also appear as inverse contrast (hypofluorescent in FAF, hyperfluorescent in NI-AF).

In the current study, areas showing hypo- or hyperfluorescence on FAF and NI-AF were considered pathologic and all of these areas were included in the measurements.

Both imaging methods yielded similar lesion progression rates and showed statistically significant increases in lesion area, indicating that NI-AF is as effective as FAF in lesion identification. Pilotto et al.^12^ also reported similar results.

In the current study, BCVA declined by a mean of 0.08±0.016 logMAR annually. Both imaging methods revealed a moderate association between lesion progression and deterioration of visual acuity and there was no statistical difference between the two associations. However, the ‘r’ value of the NI-AF values was slightly higher than that of the FAF values (r=0.362, p<0.05 and r=0.311; p<0.05, respectively) which may suggest that NI-AF measurements show the changes in visual acuity better. If NI-AF imaging is considered an indicator of RPE health, this may correspond to greater vision loss in patients with RPE atrophy.

Fleckenstein et al.^13^ studied the relationship between AMD status in one eye and geographic atrophy in the fellow eye; they found that higher atrophy progression rate in fellow eyes corresponded to early or moderate AMD. Sunness et al.^14^ compared both eyes of patients with each other and found higher progression rates in patients with larger areas of geographic atrophy.

In our search of the literature we found no studies comparing lesion size and progression rate in the same eyes. Therefore, we believe our study contributes to the literature in this respect. In this study we demonstrated that rates of lesion progression and visual acuity loss are statistically significantly higher in eyes with larger initial lesion size compared with eyes with smaller lesions.

In summary, large lesions observed with both FAF and NI-AF show faster progression than smaller lesions, and lesion growth rate increases as the lesions increase in size. Naturally, the rate of vision loss changes in proportion to the lesion progression rate.

The above-mentioned lesion characteristics can present in both imaging methods as increased and/or decreased autofluorescent features that vary based on AMD grade. This also suggests it may be the yet unknown mechanism in the pathophysiology of AMD. Furthermore, it is known that NI-AF is superior to FAF for the early detection of foveal and parafoveal lesions in particular due to foveal pigment shadowing. It should be kept in mind that these two imaging methods complement, but are not alternatives, to one another.

In conclusion, the progression of AMD can be evaluated equally well by NI-AF as by FAF imaging. NI-AF imaging is a diagnostic method that, used as a complement to FAF imaging, should be taken into account when evaluating patients.

## Figures and Tables

**Table 1:**
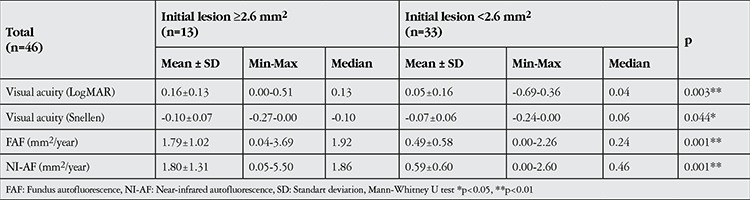
Evaluation of lesion progression rates based on initial lesion size (≥2.6 mm2; approximately 1 disc area)
